# Impact of Hypoventilation Training on Muscle Oxygenation, Myoelectrical Changes, Systemic [K^+^], and Repeated-Sprint Ability in Basketball Players

**DOI:** 10.3389/fspor.2020.00029

**Published:** 2020-04-03

**Authors:** Julien Lapointe, Pénélope Paradis-Deschênes, Xavier Woorons, Fréderic Lemaître, François Billaut

**Affiliations:** ^1^Département de Kinésiologie, Université Laval, Quebec City, QC, Canada; ^2^University of Lille, University of Artois, University of Littoral Côte d'Opale, ULR 7369 - URePSSS - Unité de Recherche Pluridisciplinaire Sport Santé Société, Lille, France; ^3^Faculté des Sciences du Sport, Université de Rouen, Rouen, France

**Keywords:** repeated-sprint ability, breath-hold, hypoxia, hypoventilation, muscle oxygenation, muscle recruitment, potassium

## Abstract

This study investigated the impact of repeated-sprint (RS) training with voluntary hypoventilation at low lung volume (VHL) on RS ability (RSA) and on performance in a 30-15 intermittent fitness test (30-15_IFT_). Over 4 weeks, 17 basketball players included eight sessions of straight-line running RS and RS with changes of direction into their usual training, performed either with normal breathing (CTL, *n* = 8) or with VHL (*n* = 9). Before and after the training, athletes completed a RSA test (12 × 30-m, 25-s rest) and a 30-15_IFT_. During the RSA test, the fastest sprint (RSA_best_), time-based percentage decrement score (RSA_Sdec_), total electromyographic intensity (RMS), and spectrum frequency (MPF) of the biceps femoris and gastrocnemius muscles, and biceps femoris NIRS-derived oxygenation were assessed for every sprint. A capillary blood sample was also taken after the last sprint to analyse metabolic and ionic markers. Cohen's effect sizes (ES) were used to compare group differences. Compared with CTL, VHL did not clearly modify RSA_best_, but likely lowered RSA_Sdec_ (VHL: −24.5% vs. CTL: −5.9%, group difference: −19.8%, ES −0.44). VHL also lowered the maximal deoxygenation induced by sprints ([HHb]_max_; group difference: −2.9%, ES −0.72) and enhanced the reoxygenation during recovery periods ([HHb]_min_; group difference: −3.6%, ES −1.00). VHL increased RMS (group difference: 18.2%, ES 1.28) and maintained MPF toward higher frequencies (group difference: 9.8 ± 5.0%, ES 1.40). These changes were concomitant with a lower potassium (K^+^) concentration (group difference: −17.5%, ES −0.67), and the lowering in [K^+^] was largely correlated with RSA_Sdec_ post-training in VHL only (*r* = 0.66, *p* < 0.05). However, VHL did not clearly alter PO_2_, hemoglobin, lactate and bicarbonate concentration and base excess. There was no difference between group velocity gains for the 30-15_IFT_ (CTL: 6.9% vs. VHL: 7.5%, ES 0.07). These results indicate that RS training combined with VHL may improve RSA, which could be relevant to basketball player success. This gain may be attributed to greater muscle reoxygenation, enhanced muscle recruitment strategies, and improved K^+^ regulation to attenuate the development of muscle fatigue, especially in type-II muscle fibers.

## Introduction

As in most team sports, basketball players have to perform various efforts including sprinting, jumping, and shuffling interspersed with relatively short and active periods of recovery (i.e., running and walking). To enhance fitness and delay neuromuscular fatigue, conditioning programs revolve around the determinants of repeated-sprint (RS) ability (RSA). These include energetic substrate depletion, metabolite accumulation, ionic and muscle excitability changes, and altered muscle recruitment strategies (Billaut and Bishop, 2009; Girard et al., 2011). In this never-ending quest for training optimization, the use of extreme environments has become very popular to increase the stress placed on athletes. Performing RS training in hypoxia (the so-called RSH modality) can enhance some peripheral limiting factors of RSA and improve the ability to repeat all-out efforts (i.e., sprint endurance) more than the same training performed in normoxia (Billaut et al., 2012; Brocherie et al., 2017). Among these purported factors, the enhancement of oxygen delivery to and oxygenation of active skeletal muscles and fast-twitch fibers recruitment have been demonstrated on several occasions and in various sport modalities (Faiss et al., 2013).

However, attending a training camp at terrestrial altitude and/or using hypoxic generators require specific logistic and equipment which can be prohibitive. Exercising while voluntarily holding one's breath at low lung volume elicits levels of arterial blood O_2_ saturation (SpO_2_) similar to those observed during typical hypoxic conditioning programs simulating altitudes from 1,500 to 3,000 m (i.e., SpO_2_ 78–92%). Mean SpO_2_ has been shown to drop down to ~88% in young healthy adults holding their breath at functional residual capacity during moderate intensity cycling efforts (Yamamoto et al., 1987). When applied to RS exercises, voluntary hypoventilation at low lung volume (VHL) has also been reported to decrease SpO_2_ (~87%) and to lead to greater muscle deoxygenation and blood lactate accumulation when compared to the same exercise protocol with normal breathing (Woorons et al., 2017).

VHL has recently been applied to RS training, and data demonstrate promising improvements in RSA in running (Fornasier-Santos et al., 2018), swimming (Trincat et al., 2017), and cycling (Woorons et al., 2019b) after only 2–4 weeks of training. However, these studies were limited in elucidating the physiological mechanisms involved in the performance gains. So far, blood lactate concentration ([Lac^−^]) has been shown to increase after VHL training at all-out intensity (Trincat et al., 2017; Woorons et al., 2019b). This adaptation is in line with the observation of greater acute [Lac^−^] after a single RS protocol and typically reflects a higher contribution from the anaerobic glycolysis pathway (Woorons et al., 2017). A greater systemic O_2_ consumption has also been observed and ascribed to an increase in stroke volume subsequent to ventricular diastolic filling in response to the large and abrupt inspiration taken immediately at the end of a breath-hold (Woorons et al., 2019b). However, other important limiting factors of RSA have not yet been fully explored. For example, enhanced blood flow to and reoxygenation of skeletal muscles, improved muscle recruitment strategies and reduced perturbations in muscle transmembrane sodium (Na^+^) and potassium ions (K^+^) concentration gradients, which are known to be readily affected by training in hypoxia, could each contribute to improving RSA. Furthermore, team-sport athletes often perform sprints with changes of direction (COD) in their training. While VHL has been successfully implemented during acute exercise with COD (Woorons et al., 2019a), the eccentric phase induced by repeated COD over weeks of training may increase muscle damages and/or neuromuscular fatigue (Chaabene et al., 2018). This approach therefore needs to be tested before recommending implementation within daily practice.

Therefore, the aim of this study was to examine additional mechanisms underlying the enhancement of sprint performance after VHL training. We also aimed to examine whether this approach could be feasible at all-out intensity with COD, as performed regularly in team sports. Based on the impact of RSH training on physiological responses, we hypothesized that VHL training would improve blood volume and muscle reoxygenation, enhance acid-base balance and K^+^ regulation, and maintain muscle recruitment.

## Materials and Methods

### Participants

Seventeen athletes (5 women and 12 men) were recruited from the Laval University basketball club (mean ± SD; age, 22.3 ± 1.2; height, 185.5 ± 11.7 cm; weight, 88.6 ± 16.9 kg). Players competed at national level, and training volume at the time of the study was 8 sessions per week for an average of ~13 h. All participants were healthy, non-smokers, did not use any medication, and were asked to avoid vigorous exercise, alcohol and caffeine 24 h before every test and to maintain their regular diet throughout the study. The study was approved by the ethics committee of Laval University, and the experiment was conducted in accordance with the principles established in the Declaration of Helsinki.

### Experimental Design

The study took place during the preparation phase to competition in May and was integrated within the annual strength and conditioning planning to limit methodological invalidity. The experimental protocol consisted of two testing sessions performed before (Pre-) and after (Post-) a 4-week RS-specific training (i.e., 8 training sessions). The testing session at Post- was conducted 3–4 days after the last training session. The two testing sessions consisted of a running RSA and a 30-15_IFT_ tests separated by 2 days without intensive training. Before initial testing, participants visited the laboratory for two familiarization sessions. In the first session, resting heart rate, blood pressure, height and weight were recorded. Then, participants were familiarized with the VHL technique for about 30 min. This technique has been fully described by Woorons et al. (2017). Briefly, it consists of breath-holding episodes at low lung volume performed during brief repeated maximal sprints. Immediately before every sprint, participants were asked to exhale down to functional residual capacity and to hold their breath while running as fast as they could for the duration of the sprint (6-s). Right after the sprint, a second exhalation was performed in order to evacuate the carbon dioxide accumulated in lungs. The experimenters observed the breathing patterns and gave constant feedback on the technique. In addition, they questioned the participants on the difficulty of applying the technique during sprints. When subjects were able to complete a 6-s sprint at maximum velocity while properly performing the breathing technique, familiarization was considered complete. The same familiarization protocol was undertaken with the COD. In the second session participants were familiarized with the 30-15_IFT_ test and again with the VHL technique. After the first testing session (Pre-), participants were matched into pairs based on their performances during the RS (score decrement) and 30-15_IFT_ tests (total distance), and then randomly assigned to a group that performed the RS training with normal breathing (CTL, *n* = 8) or with the VHL technique (VHL, *n* = 9).

### Repeated-Sprint Training

**Figure 1:**
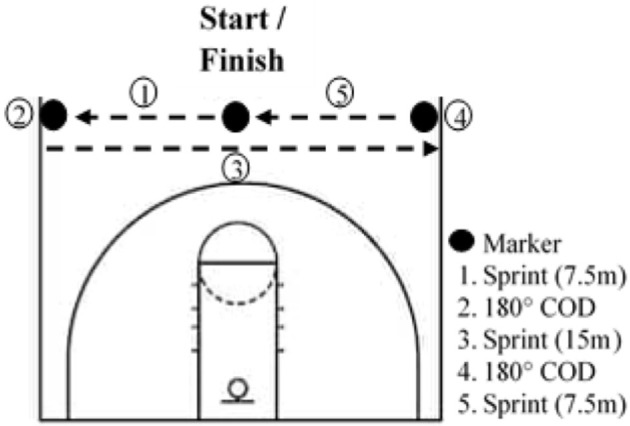
Layout of repeated sprint exercise with changes of direction (COD) on a basketball court.

Athletes had to complete 8 RS training sessions over 4 weeks, with at least 2 days between sessions. Every training session was preceded by a standardized warm-up including active mobility, dynamic stretches, various activation exercises for the posterior chain and ankle, and ended with two 20-m sprints. The first session of the week always included COD and was conducted on an indoor basketball field. Participants started in the middle of the basketball court facing the baseline, and after a count-down they had to touch one sideline after the other and finish where they started (Figure 1). At each repetition, the start was in the other direction. The second session of the week was conducted outdoors on an American football field and did not include COD. In both training protocols, participants had to sprint for 6 s and then had 24 s of semi-active recovery (i.e., walking to the started line). For the COD protocol, participants were asked to perform the maximum of the running pattern in 6 s. During the linear running protocol, participants had to run the maximal distance in 6 s. This work-to-rest ratio has been documented in previous studies (Woorons et al., 2017). Participants performed 3 sets of 6 sprints in the first and last week and 3 sets of 8 sprints in the second and third week. Each set was separated by a 3-min semi-active recovery. We chose to increase the training volume in the second and third weeks of training in order to have a greater training load. On the other hand, the training volume was reduced in the last week to avoid, or at least limit, fatigue in the perspective of the Post-testing session, as usually performed in sport settings. Both groups performed the same training sessions, but the CTL kept a normal breathing pattern. Every session was supervised to ensure that the VHL technique was correctly applied. SpO_2_ was randomly assessed in both groups throughout the training sessions with a finger pulse oximeter (Nonin Oxywatch, accuracy at 70–100%: ~2%).

### Repeated-Sprint Ability Test

The RSA pre- and post-tests were performed on an indoor American football field. The protocol consisted of 12 × 30 m straight-line running sprints interspersed with 20 s of active recovery (run back to the start line). Participants were instructed to sprint as fast as possible during every sprint with no pacing strategy and to breathe normally. Participants assumed a standardized starting position with the dominant leg in front and a two-point stance. Strong verbal encouragements were provided. Before the test, participants performed a standardized warm-up after which electromyographic (EMG) and near-infrared spectroscopy (NIRS) probes were installed (see sections below).

A photodetector (SciencePerfo, Quebec, Canada; 50 Hz) was used to measure sprint times. As soon as a participant started a sprint, an integrated algorithm allowed to begin the recording and prevent false start error. The photodetector recorded maximal speed, split times and speed at 5, 10, 15, and 30 m. The best sprint time (RSA_best_) and the average completion time of the 12 sprints (RSA_mean_) were computed, and time-based percent decrement score (RSA_Sdec_) was calculated as follows: [(total sprint time/ideal sprint time × number of sprint) −1] ×100 where number of sprints was 12 (Glaister et al., 2008).

The rating of perceived exertion (RPE) score was recorded directly after the last sprint using the Borg 10-point scale to assess subjective perceived exertion. A blood sample was taken 1-min post for subsequent analysis.

### 30-15 Intermittent Fitness Test

This maximal aerobic test consisted of 30-s shuttle runs interspersed with 15 s of semi-active recovery. The first stage was set at 8 km·h^−1^ and speed increased by 0.5 km·h^−1^ at every stage completed until volitional exhaustion. Tests were conducted indoor on a basketball court and participants had to shuttle run between two baselines (28 m apart). The running pace was governed by a soundtrack and participants were strongly encouraged. The test ended when a participant was not able to keep running at the imposed pace. The velocity of the last completed stage was retained as the participant V_IFT_. This test is used extensively to assess the aerobic fitness of team-sport players (Buchheit et al., 2009). The RPE score was recorded right after the last stage.

### Near-Infrared Spectroscopy Measurements

Muscle blood volume and oxygenation were assessed continuously during the RSA test using a spatially-resolved portable NIRS apparatus (PortaMon, Artinis Medical System BV, Netherlands). The NIRS device was installed on the gastrocnemius lateralis muscle belly (1/4 of the line between the head of fibula and the heel), parallel to muscle fiber direction to quantify changes in absorption of near-infrared light by oxyhemoglobin (HbO_2_) and deoxyhemoglobin (HHb). The device was enclosed in a transparent plastic bag to protect it from sweat, fixed with tape and covered by a black bandage to avoid interference with background light. The position was marked with indelible pen for the post-visit. A modified form of the Beer-Lambert law, using two continuous wavelengths (760 and 850 nm) and a differential optical path length factor of 4.95, was used to calculate micromolar changes in tissue HbO_2_ (Δ[HbO_2_]), HHb (Δ[HHb]), and total hemoglobin [Δ[tHb] = [HbO_2_] + [HHb]; used as an index of change in regional blood volume (van Beekvelt et al., 2001)].

The NIRS data were acquired at 10 Hz and then filtered using a tenth-order Butterworth low-pass filter with a 4 Hz cut-off frequency. Analysis of muscle O_2_ extraction was limited to [HHb] because this variable is less sensitive than [HbO_2_] to perfusion variations and abrupt blood volume changes during contraction and recovery (de Blasi et al., 1993; Ferrari et al., 2004). From the filtered signal, one value for each of the maximal and minimal [HHb] and [tHb] was manually identified for every sprint/recovery cycle throughout the RSA test for accurate detection of oxygenation peaks and nadirs (Faiss et al., 2013; Rodriguez et al., 2018). All peaks ([HHb]_max_ and [tHb]_max_), nadirs ([HHb]_min_ and [tHb]_min_), and amplitude changes (i.e., peak-to-nadir difference: Δ[HHb] and Δ[tHb]) were then normalized to the peak, nadir and amplitude recorded during the first sprint/recovery cycle (Faiss et al., 2013).

### Electromyographic Acquisition and Analysis

During every sprint of the RSA test, the EMG signals of the biceps femoris (BF) and gastrocnemius lateralis (GAS) were recorded from the dominant leg with surface electrodes (Delsys, Trigno Wireless, Boston, MA). Electrode sites were prepared before every test (hair shaved, skin lightly abraded and cleaned with alcohol). Electrodes were fixed longitudinally over the muscle belly according to SENIAM's recommendations (Hermens et al., 2000). The position was marked with indelible pen for the post-visit and participants were asked to maintain the writing visible on the skin. The EMG signal was pre-amplified and filtered (bandwidth 12–500 Hz, gain = 1,000, sampling frequency 2 kHz) and recorded with Delsys hardware (Bagnoli EMG System; Delsys, Inc., USA). The activity of each muscle was determined by measuring the mean value of the root-mean-square (RMS) and the median power frequency (MPF) between the onset and the offset of the first 6 subsequent bursts of the sprint. The RMS and MPF values of both muscles were summed and then normalized to the first sprint value of each condition (Smith and Billaut, 2010).

### Blood Sampling and Analysis

A 92-μL blood sample was drawn ~1 min after the last sprint from fingertips using a capillary tube and analyzed with a portable blood analyser (Epoc^®^ Blood Analysis System, Siemens Healthinners, Munich, Germany). A thermal quality assurance calibration was conducted before the pre- and the post-session with a buffered aqueous solution according to manufacturer's recommendations. The blood measured pH, carbon dioxide and oxygen partial pressure (PCO_2_, PO_2_), concentrations of sodium ([Na^+^]), potassium ([K^+^]), ionized calcium ([Ca^++^]), chloride ([Cl^−^]), glucose ([Glu]), lactate ([Lac^−^]) and hematocrit (Hct), and calculated hemoglobin ([cHgb]), bicarbonate ([${\text{cHCO}}_{3^{-}}$]), total carbon dioxide, base excess of extra cellular fluid [BE(ecf)], and base excess of blood [BE(b)].

### Statistical Analysis

All data are reported as mean ± standard deviation (SD), percentage of normalized values or percentage of change from Pre-training. Before analysis, all variables were log-transformed except for negative values (base excess). The Post- to Pre-training and VHL-CTL differences were analyzed using Cohen's effect size (ES) ± 90% confidence limits and compared to the smallest worthwhile change (0.2 multiplied by the between-participant SD) (Batterham and Hopkins, 2006; Hopkins et al., 2009). Effect sizes were classified as small (>0.2), moderate (>0.5), and large (>0.8). Using mechanistic inferences, qualitative probabilistic terms for benefit were assigned to each effect using the following scale: <0.5% most unlikely; 0.5–5% very unlikely; 5–25% unlikely; 25–75% possibly; 75–95% likely; 95–99.5% very likely; >99.5% almost certainly. If the chance of having better/greater and poorer/lower performances or physiological changes were both >5%, the effect was deemed “unclear” or “unmeaningful” (Batterham and Hopkins, 2006; Hopkins et al., 2009). Pearson correlations were calculated to assess associations between physiological changes and performance improvements. Correlation coefficients of >0.1, >0.3, >0.5, and >0.7 were considered small, moderate, large and very large (Hopkins et al., 2009).

## Results

Of the 17 participants recruited, 13 completed the entire protocol and were included in the analysis (VHL, *n* = 7 and CTL, *n* = 6). One participant could not complete the study because of injury not related to the study. The three others missed more than 3 training sessions and were therefore excluded from data analyses. All participants from the VHL group tolerated the breathing technique without any issues or complications. During training, the averaged SpO_2_ recorded during the three sets (including sprints and recovery phases, but excluding the inter-set 3-min recovery) was 87.7 ± 4.6% for the VHL group and 96.9 ± 0.5% for CTL.

### Performance

**Figure 2:**
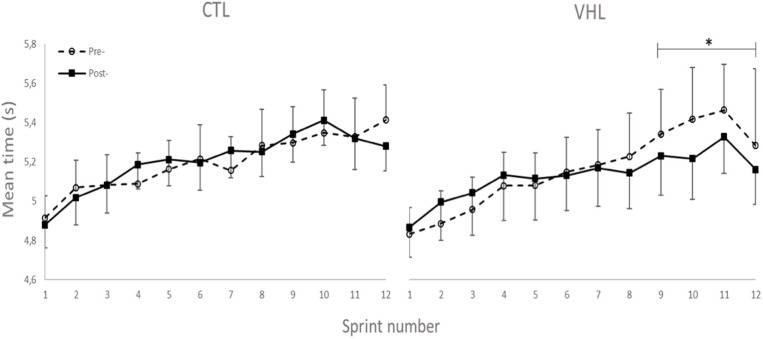
Completion time for every sprint of the RSA test performed with normal breathing (CTL) and voluntary hypoventilation at low volume (VHL) before and after 4 weeks of repeated-sprint training. Data are presented as means ± SE. ^*^Small effect between groups.

Performance results for the RSA and 30-15_IFT_ tests are displayed in Table 1. Figure 2 also depicts the completion time for every sprint in both VHL and CTL. The calculated smallest worthwhile change for RSA_best_ equated to 0.5 s. Training had no effect on RSA_best_ in any groups, but had a possible benefit on RSA_mean_ in both groups (VHL: −2.5 ± 1.8% vs. CTL: −3.4 ± 2.6%), with no clear difference between groups. However, when the last four sprints of the series were analyzed, VHL clearly reduced the mean completion time compared to CTL (−3.0 ± 4.3%, ES −0.31 ± 4.3, chances to observe poorer/trivial/better performance after VHL: 3%/30%/67%). The calculated smallest worthwhile change for RSA_Sdec_ was 0.42%. The VHL group's RSA_Sdec_ clearly improved from 7.3 ± 3.2% to 5.5 ± 2.7% with the intervention (−24.5 ± 27.2%, ES −0.47 ± 0.40), while the change in the CTL group from 7.1 ± 3.1 to 6.5 ± 2.5% remained unclear (−5.9 ± 21.5%, ES −0.13 ± 0.42). This yielded a likely small advantage for VHL over CTL (group difference: −19.8 ± 33.8%, ES −0.44 ± 0.58, 4%/20%/76%). Maximal velocity in the 30-15_IFT_ improved both in VHL (7.5 ± 2.9%) and in CTL (6.9 ± 3.4%) above the smallest worthwhile change of 0.33 km/h, with no clear difference between groups.

**Table 1:** Mean changes in performance and perceptual exercise responses in the repeated-sprint ability (RSA) and the 30-15_IFT_ tests after repeated-sprint training performed with voluntary hypoventilation at low lung volume (VHL) or normal breathing (CTL).

		**Pre-**	**Post-**	**Within group Post/Pre (Cohen's d)**	**Probability (%)**
RSA_best_ (s)	VHL CTL VHL-CTL (Cohen's) Qualitative inference	4.80 ± 0.35 4.83 ± 0.36	4.86 ± 0.36 4.88 ± 0.43 0.04 ± 0.30 Unclear	0.15 ± 0.17 0.10 ± 0.22	31/61/0 21/77/2 19/73/9
RSA_mean_ (s)	VHL CTL VHL-CTL (Cohen's) Qualitative inference	5.16 ± 0.47 5.18 ± 0.51	5.03 ± 0.41 5.01 ± 0.55 0.10 ± 0.32 Unclear	−0.27 ± 0.19 −0.32 ± 0.23	0/26/74 0/19/81 30/64/6
RSA_Sdec_ (%)	VHL CTL VHL-CTL (Cohen's) Qualitative inference	7.25 ± 3.18 7.08 ± 3.08	5.49 ± 2.70 6.46 ± 2.50 –**0.44** **±** **0.58** **Likely beneficial**	−0.47 ± 0.40 −0.13 ± 0.42	1/12/88 9/53/38 **4/20/76**
RPE RSA (AU)	VHL CTL VHL-CTL (Cohen's) Qualitative inference	7.5 ± 1.15 8.03 ± 1.23	7.08 ± 1.22 7.88 ± 1.22 −0.24 ± 0.53 Unclear	−0.32 ± 0.30 −0.11 ± 0.46	1/23/77 12/52/37 8/37/55
V_IFT_ (km·h^−1^)	VHL CTL VHL-CTL (Cohen's) Qualitative inference	18.78 ± 1.68 18.63 ± 1.75	20.18 ± 1.39 19.88 ± 1.41 0.07 ± 0.49 Unclear	0.81 ± 0.32 0.69 ± 0.35	100/0/0 98/1/0 33/50/17
RPE 30–15 (AU)	VHL CTL VHL-CTL (Cohen's) Qualitative inference	7.58 ± 0.68 7.72 ± 1.22	7.94 ± 0.76 8.38 ± 0.98 −0.33 ± 0.96 Unclear	0.43 ± 0.59 0.53 ± 0.67	76/20/4 81/15/4 17/23/57

*Data are presented as means ± SD. Cohen's effect size ± 90% confidence limits. Clear changes within and between groups are indicated in bold. RSA_best_, best sprint time; RSA_mean_, average completion time of the 12 sprints; RSA_Sdec_, % score decrement; RPE, rate of perceived exertion; V_IFT_, maximal velocity reached in the Intermittent 30–15 fitness test; AU, arbitrary units*.

### Muscle Oxygenation

**Figure 3:**
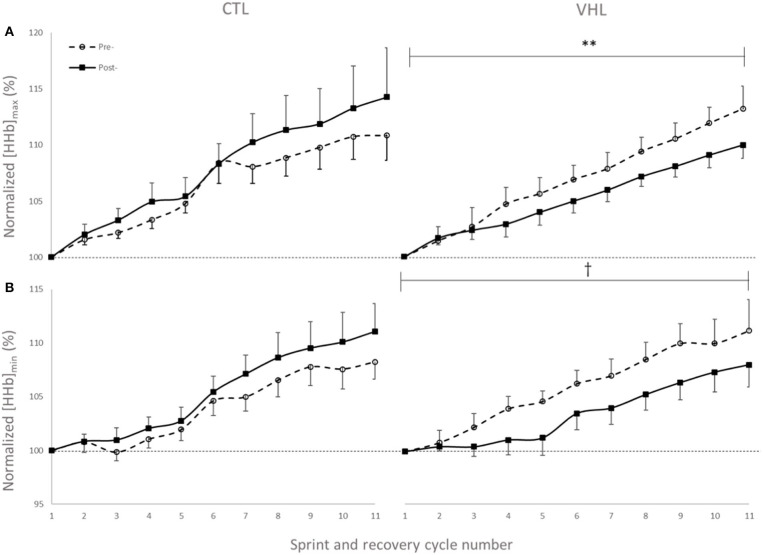
Peak **(A)** and nadir **(B)** values of normalized deoxyhemoglobin concentration ([HHb]) over 11 sprint/recovery cycles with normal breathing (CTL) and voluntary hypoventilation at low lung volume (VHL) before and after 4 weeks of training. Data are presented as means ± SD, expressed as a percent of the first sprint/recovery cycle. ^**^Moderate effect between groups; ^†^Large effect between groups.

**Figure 4:**
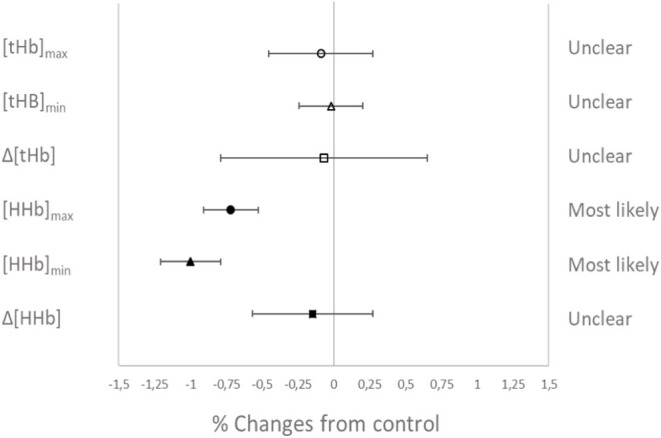
Percentage difference and qualitative interference in the change in NIRS variables from Pre- to Post- in VHL compared to CTL.

Muscle oxygenation during the RSA tests in VHL and CTL is depicted in Figure 3 and between-groups changes for NIRS variables are displayed in Figure 4. From pre- to post-training, [tHb] peaks and amplitudes did not change in either groups. However, [tHb]_min_ increased in both VHL (1.2 ± 0.3%, ES 0.31 ± 0.09, 97%/3%/0%) and CTL (1.3 ± 0.8%, ES 0.30 ± 0.19, 81%/19%/0%), with no difference between groups. Furthermore, [HHb]_max_ clearly decreased in VHL (−1.5 ± 0.6%; ES −0.39 ± 0.15, 0%/2%/98%), but clearly increased in CTL (1.4 ± 0.6%, ES 0.30 ± 0.18, 92%/8%/0%). As a result, there was an almost certain difference between groups with VHL attenuating the maximal deoxygenation (−2.9 ± 0.8, ES −0.72 ± 0.19, 0%/0%/100%). Similar changes were observed for [HHb]_min_ with a clear decrease in VHL (−2.3 ± 0.6%; ES −0.65 ± 0.17, 0%/0%/100%) and a clear increase in CTL (1.3 ± 0.5%, ES 0.33 ± 0.12, 96%/4%/0%), yielding an almost certain difference between groups (−3.6 ± 0.8%, ES −1.00 ±0.21, 0%/0%/100%).

### Electromyographic Activity

**Figure 5:**
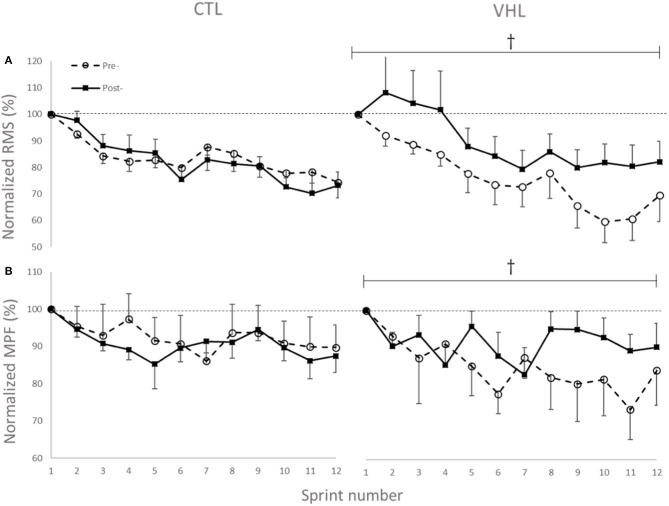
Changes in normalized EMG amplitude (RMS) **(A)** and median power frequency (MPF) **(B)** of the biceps femoris and gastrocnemius muscles during 12 sprints with normal breathing (CTL) and voluntary hypoventilation at low lung volume (VHL) before and after 4 weeks of training. Data are presented as means ± SD, expressed as a percent of sprint 1. Large effect between groups. The RMS values decreased from 0.45 ± 0.15 mV to 0.35 ± 0.13 mV in Pre- and from 0.43 ± 0.17 mV to 0.31 ± 0.11 mV in Post- in the CTL group. In VHL, RMS decreased from 0.41 ± 0.11 mV to 0.31 ± 0.14 mV in Pre- and from 0.41 ± 0.11 mV to 0.32 ± 0.14 mV in Post-. Average MPF values decreased from 105.5 ± 25.2 Hz to 99.2 ± 23.8 Hz in Pre- and from 104.6 ± 25.3 Hz to 91.4 ± 25.4 Hz in Post- in CTL group. In VHL, MPF decreased from 112.3 ± 17.0 Hz to 108.4 ± 16.3 Hz in Pre- and from 106.6 ± 17.4 Hz to 100.1 ± 18.1 Hz in Post-.

The changes in temporal profile of EMG amplitude (RMS) and spectral profile of median power spectrum (MPF) for the two investigated muscles in both conditions are displayed in Figure 5. As depicted, and in line with other RSA studies, the EMG RMS decreased over the 12 sprints both pre- and post-training. However, while the average value for the 12 sprints did not change in CTL (−1.5±2.8, ES−0.14 ± 0.26, 2%/64%/34%), RMS almost certainly increased in VHL (16.5 ± 4.5%, ES 1.01 ± 0.29, 100%/0%/0%). This led to an almost certain difference between training groups (18.2 ± 5.1%, ES 1.28 ± 0.38, 100%/0%/0%). Similar to the RMS changes, MPF decreased from Pre- to Post-training in CTL (−2.0 ± 2.0%, ES −0.45 ± 0.43, 1%/15%/84%), but increased in VHL (7.7 ± 4.7%, ES 0.97 ± 0.61, 98%/2%/0%). There was a very likely clear advantage of VHL training in maintaining MPF over CTL (9.8 ± 5.0%, ES 1.40 ± 0.74, 99%/1%/0%).

### Blood Sample Analysis

Table 2 displays the changes and VHL-CTL differences for blood parameters in the RSA tests. In Post-, compared to Pre-, pH increased in CTL (0.5 ± 0.5%; ES 0.58 ± 0.58, 88%/10%/2%), but remained unchanged in VHL, yielding a clear difference between groups (−0.4 ± 0.5%, ES −0.38 ± 0.51, 4%23%/73%). Partial pressure of carbon dioxide (PCO_2_) marginally decreased in CTL (−3.6 ± 5.5%; ES −0.27 ± 0.40, 3%/34%/63%), and remained unchanged in VHL, yielding a likely difference between groups (8.5 ± 11.2%, ES 0.61 ± 0.79, 82%/14%/5%). Although the changes in [K^+^] were not meaningfully different from Pre- to Post- within each group (10.4% increase in CTL vs. 9.0% decrease in VHL), comparing the changes between groups yielded a likely difference (−17.5 ± 31.2, ES −0.67 ± 0.94, 5%/13%/80%). The lowering in [K^+^] was largely correlated with the improvement in RSA_Sdec_ post-training in the VHL group only (*r* = 0.66, *P* = 0.03). All other blood markers were not meaningfully different between training groups.

**Table 2:** Mean changes in blood parameters following the RSA test after repeated-sprint training performed with voluntary hypoventilation at low lung volume (VHL) or normal breathing (CTL).

	**CTL**		**VHL**		**Difference between groups**		
	**Pre-**	**Post-**	**Pre-**	**Post-**	**% D**	**Cohen's**	**Probability (%)**
pH	7.22 ± 0.04	7.25 ± 0.06	7.22 ± 0.08	7.23 ± 0.09	−0.4 ± 0.5	–**0.38** **±** **0.14**	4/23/73
PCO_2_ (mmol·L–^1^)	31.95 ± 3.10	30.88 ± 3.85	31.36 ± 5.11	32.46 ± 2.60	8.5 ± 11.2	**0.61** **±** **0.79**	82/14/5
PO_2_ (mmol·L–^1^)	88.53 ± 2.33	88.85 ± 5.04	92.64 ± 11.60	89.81 ± 8.34	−3.0 ± 7.7	−0.33 ± 0.8	13/26/61
[Na^+^] (mmol·L^−1^)	144.00 ± 1.67	144.67 ± 2.25	144.75 ± 4.68	144.00 ± 1.77	−0.9 ± 2.5	−0.44 ± 1.15	17/19/65
[K^+^] (mmol·L^−1^)	5.02 ± 0.3	5.67 ± 1.33	6.73 ± 2.64	5.93 ± 1.41	−17.5 ± 31.2	**−0.67** **±0.94**	5/13/80
[Ca^++^] (mmol·L^−1^)	1.25 ± 0.02	1.25 ± 0.05	1.29 ± 0.08	1.29 ± 0.06	−0.7 ± 4.4	−0.13 ± 0.86	25/30/44
[Cl^−^] (mmol·L^−1^)	112.83 ± 2.56	113.00 ± 3.58	114.00 ± 6.57	113.75 ± 4.17	−0.3 ± 5.3	−0.06 ± 1.25	36/22/42
[Glu] (mmol·L^−1^)	8.53 ± 1.27	8.35 ± 1.22	7.49 ± 0.89	7.33 ± 0.91	−0.2 ± 4.7	−0.01 ± 0.30	12/74/14
[Lac^−^] (mmol·L^−1^)	13.49 ± 1.51	12.12 ± 1.53	14.03 ± 2.38	12.98 ± 2.97	2.1 ± 12.4	0.11 ± 0.63	40/40/19
Hct (%)	45.67 ± 2.94	47.33 ± 3.93	46.13 ± 5.94	46.88 ± 4.70	−1.5 ± 4.4	−0.15 ± 0.41	8/51/41
[${\text{cHCO}}_{3^{-}}$] (mmol·L^−1^)	13.05 ± 1.99	13.65 ± 1.78	12.98 ± 2.92	13.79 ± 2.72	1.7 ± 13.7	0.09 ± 0.69	39/38/23
[cTCO_2_] (mmol·L^−1^)	14.02 ± 2.04	14.58 ± 1.82	13.93 ± 2.99	14.78 ± 2.72	2.2 ± 13.8	0.12 ± 0.73	42/36/21
BE(ecf) (mmol·L^−1^)	−14.70 ± 2.54	−13.52 ± 2.48	−14.74 ± 3.86	−13.79 ± 4.07	−0.2 ± 1.9	−0.07 ± 0.55	20/47/33
BE(b) (mmol·L^−1^)	−13.47 ± 2.45	−12.10 ± 2.51	−13.46 ± 3.75	−12.61 ± 4.07	−0.5 ± 1.8	−0.15 ± 0.53	13/43/43
SpO_2_ (%)	94.85 ± 0.45	95.35 ± 0.89	95.28 ± 1.44	95.14 ± 0.75	−0.7 ± 1.1	−0.61 ± 1.04	9/15/76
cHgb (g·dL^−1^)	15.48 ± 0.96	16.05 ± 1.30	15.65 ± 2.03	15.83 ± 1.59	−2.0 ± 4.5	−0.20 ± 0.43	6/44/49

*Data are presented as means ± SD. Cohen's effect size ± 90% confidence limits. Clear changes between groups are indicated in bold*.

### Perceptual Exercise Responses

RPE scores for the RSA and 30-15_IFT_ tests are displayed in Table 1. RPE only decreased after VHL in Post- compared with Pre- in the RSA test (−5.8 ± 5.7%, ES −0.32, 1%/23%/77%), while CTL did not exhibit any changes. However, there was no clear difference between groups. In the 30-15_IFT_ test, RPE increased in both groups at Post-, and there was no difference between groups.

## Discussion

We report that 8 sessions of RS training including COD performed with voluntary respiratory blockage at low lung volumes by basketball players elicited clear, though not large, RSA gain compared to training with unrestricted breathing, but this improvement was not transferable to longer activities. The novel findings were that training with VHL, in contrast to control, enhanced muscle reoxygenation during recovery periods, increased total electric activity (RMS) and power spectrum frequency (MPF) of the lower-limb muscles and reduced the extracellular [K^+^], which was significantly correlated with the gain in RSA.

The improvement in RSA (and absence of change in maximal speed) was also demonstrated in highly-trained rugby players who increased the maximum number of sprints before exhaustion by 64% (Fornasier-Santos et al., 2018) and in trained swimmers who displayed 35% improvement in sprint number (Trincat et al., 2017). Woorons et al. (2019b) were the first to use a close-loop design and reported a 4.1% improvement in mean power score decrement during ten 6-s sprints. In a recent study, which also used a close-loop test, running RSA was improved by 2.5% in team-sport players after 3 weeks of high-intensity cycle training with VHL (Woorons et al., 2020). Our present results confirm these findings and together robustly highlight that RSA can be improved in a relatively short timeframe within the training calendar. In most team sports, the ability of players to resist and/or limit neuromuscular fatigue to maintain the highest intensity or velocity is paramount. For instance, RSA may be decisive in the final stages of the game by giving the possibility to win possession of the ball and increasing the chances of scoring while preventing the opponents to do so (Billaut and Bishop, 2009; Girard et al., 2011). In this perspective, it is interesting to note that, so far, all studies combining VHL with RS training (including the present one), reported significant gains in RSA (Trincat et al., 2017; Fornasier-Santos et al., 2018; Woorons et al., 2019b). Conversely, RS training studies using simulated hypoxia (the RSH modality) reported either positive gains (Faiss et al., 2013, 2015) or no meaningful change (Gatterer et al., 2014; Brocherie et al., 2017). However, it is noteworthy that the RS training with VHL did not lead to greater improvement in a longer activity than CTL, since maximal aerobic performance in the 30-15_IFT_ was improved similarly in both groups.

The efficacy of VHL training has mainly been ascribed to a larger contribution from the anaerobic glycolysis (Trincat et al., 2017). In the present study, the RSA gains in VHL were concomitant with clear changes in muscle oxygenation patterns. Peaks and nadirs of the [HHb] signal were clearly lower after training in the VHL group only. The lower [HHb]_max_ indicates a lower O_2_ extraction at the muscle level during the sprints and may suggest (when analyzed in conjunction with better RSA) a metabolic shift toward anaerobic activity to produce ATP and sustain mechanical power (Woorons et al., 2011, 2017). This superior glycolytic activity should have resulted in larger lactate accumulation in the blood after the sprints. However, like others (Fornasier-Santos et al., 2018; Woorons et al., 2019b), we only reported trivial changes in [Lac^−^] between groups. The discrepancy between studies may be related to exercise duration and subsequent contribution from energy systems. While Trincat et al. (2017) used 25-m swim sprints of ~14-s, we and others (Fornasier-Santos et al., 2018; Woorons et al., 2019b) investigated running sprints of ~5-s. Longer sprints typically require greater contribution from the lactic glycolysis, probably explaining the higher [Lac^−^] post-training. It may also be caused by a greater clearance during recovery phases between sprints.

We observed a lower [HHb]_min_ during recovery periods after VHL training, whereas it increased in CTL, showing the very different effects of the two training regimens. This indicates a better reoxygenation capacity between sprints (Billaut and Buchheit, 2013) after VHL training. Such local adaptation in active locomotor muscles is reported here for the first time in the scientific literature and may explain the greater systemic VO_2_ observed during recovery phases between repeated sprints after VHL training (Woorons et al., 2019b). Alternatively, the greater muscle reoxygenation could be the consequence of greater cardiac output and arterial inflow to the muscles purported to occur after VHL (Woorons et al., 2019b). This assumption is supported by a recent study which shows that a high-intensity VHL training in cycling induces transferable benefits for running RSA (Woorons et al., 2020). Whatever the case, these avenues will have to be disentangled in future investigations. Surprisingly however, that latter study did not observe any alteration of muscle reoxygenation during a similar repeated-sprint training intervention. The difference between these findings may be explained by the methodological analysis. While the authors examined averages in the [HHb] signal over several seconds, we determined peaks and nadirs from single values which more accurately detects maximal metabolic perturbations (Rodriguez et al., 2018). The different physiological profiles of the athletes volunteering in the two studies (team-sport athletes vs. endurance cyclists) and the exercise mode (running vs. cycling) might also have come into play. Nonetheless, we interpret our data to suggest that the better reoxygenation facilitated the resynthesis of phosphocreatine (PCr) during short recovery periods (McMahon and Jenkins, 2002). This hypothesis could explain the gain in RSA without concomitant changes in the typical markers of “lactic” metabolism ([Lac^−^] and [${\text{HCO}}_{3^{-}}$]). It is further supported by the fact that PCr availability is highly critical to RSA and that aerobic oxidations and PCr become the major sources of energy as sprints are repeated while anaerobic glycolysis contribution progressively fades (for review see Billaut and Bishop, 2009; Girard et al., 2011). In addition, RSH training with 14.5% O_2_ leading to similar hypoxemia has been shown to significantly increase the intramuscular PCr content as measured with P-magnetic resonance spectroscopy in sprinters compared to training in normoxia (Kasai et al., 2019). Unfortunately, P-MRS does not allow distinguishing between fiber types, and it is currently unknown whether type-II fibers better adapt to VHL than type-I fibers (as might be the case in RSH, Faiss et al., 2013, 2015). Nonetheless, knowing that fast-twitch fibers are fully recruited at all-out intensity and that, in the present study, the EMG power spectrum was maintained toward higher stimulation frequencies (see EMG section below), we could reasonably propose that fast-twitch fibers preferentially benefited from the better reoxygenation during recovery and maintained a relatively high contribution to mechanical power in latter sprints. In fact, the cumulated completion time of the last 4 sprints was clearly lower after training in VHL (Figure 2), supporting the hypothesis of a delayed fatigue. Further investigations using magnetic resonance imaging or muscle biopsy are required to assess fiber-specific intramuscular PCr content and resynthesis rate after VHL training to support this adaptative mechanism.

In the RSA literature, reductions in EMG-derived indices RMS and MPF have been widely described (Billaut and Bishop, 2009; Girard et al., 2011) and are typically taken as reduction in total motor unit recruitment and increased reliance on slow, fatigue-resistant type-1 motor units, respectively, due to the development of neuromuscular fatigue. There is no data on electromyographic behavior of active skeletal muscles after breath-hold training, so the current study highlights for the first time the marked impact of VHL training on these neural strategies (Figure 5). While training with normal breathing did not change muscle recruitment patterns, training with VHL led to a better maintenance of the initial muscle activity over subsequent sprints (+16.5% RMS) and the recruitment of higher-frequency motor units (+7.7% MPF) concomitant to enhanced sprint endurance in later repetitions. The most likely explanation would be that the better reoxygenation during recovery phases improved the metabolic milieu of contracting muscles and attenuated the reflex inhibition originating from group III and IV afferents, thereby maintaining neural drive to skeletal muscles (Amann and Dempsey, 2008).

Another speculative alternative, which will need to be examined in future studies, may include the following. Breath-hold exercise induces a sharp accumulation of CO_2_ in blood and tissues (Woorons et al., 2017), which is a potent signaling molecule. Although the direct effects (if any) of hypercapnia on central motor command are unknown in exercising humans, an increase in arterial CO_2_ increases cerebral blood flow (Hoiland et al., 2019) which could alter the central motor command (Nybo and Rasmussen, 2007). Greater increases in cerebral blood flow have been observed during apnea in breath-hold divers than in controls and interpreted as a protection of the brain against the alteration of blood gas (Joulia et al., 2009). However, we must remember that cerebrovascular reactivity to CO_2_ is a highly modifiable response that may be altered by hypoxia, changes in blood pressure, and exercise intensity (Hoiland et al., 2019).

While changes in EMG indices may be used as surrogates of neural drive, they can also be influenced by sarcolemma excitability and thereby reflect changes in conduction velocity of action potentials. Membrane excitability is impaired during intense fatiguing exercise as a result of a lower activity of the sodium(Na^+^)/potassium(K^+^)-adenosine triphosphatase (NKA) activity to maintain transmembrane ionic gradient caused by the decline in pH and accumulation of inorganic phosphates. This ultimately results in a K^+^ ion efflux out of the interstitium that impairs peripheral contractile function (Juel et al., 2000; Fraser et al., 2002). Interestingly, we observed a lower capillary blood [K^+^] after training in the VHL group only, which was significantly correlated with the enhancement in RSA. This is the first report of ionic concentrations after VHL training, and we may speculate that the combination of training at very high intensity with respiratory blockage at low lung volume creates favorable conditions for metabolic by-products accumulation and ionic perturbations, both of which are potent stimuli for promoting adaptations in skeletal muscle K^+^ regulation (Christiansen, 2019). Along this line of reasoning, Christiansen et al. (2019) demonstrated that high-intensity interval training with blood-flow restriction (leading to 90% tissue deoxygenation assessed via NIRS during complete arterial occlusion) reduces the net K^+^ release from contracting muscles during intense exercise, due to a training-induced increase in Na^+^, K^+^-ATPase-isoform abundance in the sarcolemma and T-tubuli. Near-maximal levels of muscle deoxygenation have also been reported during repeated sprints with VHL (Woorons et al., 2017), suggesting that the current VHL training probably led to similar metabolic perturbations conducive to K^+^ regulation improvement. The re-establishment of the transmembrane K^+^ gradient could also explain in part the higher RMS and MPF observed after VHL training in the present study. However, we must acknowledge that systemic K^+^ levels may inaccurately reflect locomotor muscle K^+^ homeostasis (Juel et al., 2000), indicating the need to directly assess K^+^ efflux from exercising musculature to clarify the role of VHL with respect to regulating K^+^ homeostasis in human skeletal muscle.

Future experiments will need to ascertain some of these findings to distinguish the mechanisms underlying VHL from other hypoxic training methods and, potentially, to explore combination of modalities.

## Conclusion

The current study demonstrated that 8 sessions of VHL training including changes of direction enhanced performance during straight-line repeated sprints more than the same training with unrestricted breathing. Such training strategy could therefore be implemented in various sports settings as a practical way to induce arterial hypoxemia. Physiological responses measured after training suggested that the gain in RSA may be attributed to greater muscle reoxygenation, enhanced muscle recruitment strategies, and improved K^+^ regulation.

## Data Availability Statement

All datasets generated for this study are included in the article/supplementary material.

## Ethics Statement

The studies involving human participants were reviewed and approved by ethics committee of Laval University. The patients/participants provided their written informed consent to participate in this study.

## Author Contributions

JL and FB conceived and designed the experiments and analyzed the data. JL and PP-D performed the experiments. JL, FB, XW, and FL interpreted the results of research. JL, FB, PP-D, XW, and FL critically revised the paper and approved the final version of manuscript.

### Conflict of Interest

The authors declare that the research was conducted in the absence of any commercial or financial relationships that could be construed as a potential conflict of interest.
